# Slidable and Highly Ionic Conductive Polymer Binder for High‐Performance Si Anodes in Lithium‐Ion Batteries

**DOI:** 10.1002/advs.202205590

**Published:** 2022-12-23

**Authors:** Yifeng Cai, Caixia Liu, Zhiao Yu, Wencan Ma, Qi Jin, Ruichun Du, Bingyun Qian, Xinxin Jin, Haomin Wu, Qiuhong Zhang, Xudong Jia

**Affiliations:** ^1^ Key Laboratory of High‐Performance Polymer Material and Technology of MOE Department of Polymer Science and Engineering School of Chemistry and Chemical Engineering Nanjing University Nanjing 210023 P. R. China; ^2^ Department of Chemical Engineering Stanford University Stanford CA 95403 USA; ^3^ State Key Laboratory of Coordination Chemistry Nanjing University Nanjing 210023 P. R. China

**Keywords:** Li‐ion battery, polymer binders, polyrotaxane, silicon anode, single‐ion conductors

## Abstract

Silicon is expected to become the ideal anode material for the next generation of high energy density lithium battery because of its high theoretical capacity (4200 mAh g^−1^). However, for silicon electrodes, the initial coulombic efficiency (ICE) is low and the volume of the electrode changes by over 300% after lithiation. The capacity of the silicon electrode decreases rapidly during cycling, hindering the practical application. In this work, a slidable and highly ionic conductive flexible polymer binder with a specific single‐ion structure (abbreviated as SSIP) is presented in which polyrotaxane acts as a dynamic crosslinker. The ionic conducting network is expected to reduce the overall resistance, improve ICE and stabilize the electrode interface. Furthermore, the introduction of slidable polyrotaxane increases the reversible dynamics of the binder and improves the long‐term cycling stability and rate performance. The silicon anode based on SSIP provides a discharge capacity of ≈1650 mAh g^−1^ after 400 cycles at 0.5C with a high ICE of upto 92.0%. Additionally, the electrode still exhibits a high ICE of 87.5% with an ultra‐high Si loading of 3.84 mg cm^−2^ and maintains a satisfying areal capacity of 5.9 mAh cm^−2^ after 50 cycles, exhibiting the potential application of SSIP in silicon‐based anodes.

## Graphical abstract

A highly ionic conductive polymer binder with molecular dynamics is designed and exhibits both excellent mechanical performance and satisfactory electrochemical properties. Based on the novel multi‐functional binder, the silicon anode shows reliable performance with a high ICE and reliable performance.

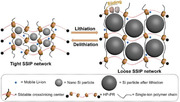

## 1 Introduction

The anode of commercial lithium‐ion batteries almost completely uses graphite as the active material, whose theoretical capacity is 372 mAh g^−1^.^[^ ^1^ ^]^ Due to high initial coulombic efficiency (ICE), excellent cycling stability, and limited volume change during charging and discharging, natural graphite and artificial graphite accounted for about 98% of the commercial anode shipments in 2021, firmly occupying the current lithium‐ion battery market.^[^ ^2^ ^]^ With the continuous technological optimization of graphite anode, its actual capacity is reaching the theoretical value, and therefore, it is difficult to make a breakthrough in the energy density of power batteries based on graphite anode. Silicon, the second most abundant element on the earth, has a high theoretical specific capacity (3579 mAh g^−1^ for Li_15_Si_4_ and 4200 mAh g^−1^ for Li_22_Si_5_) and a low working voltage (≈0.4 V vs Li/Li^+^). It is expected to replace graphite as the next generation anode material for high energy density lithium‐ion batteries.^[^ ^3^ ^]^ However, the large volume change (volume expansion rate over 300% after lithiation) will result in fragmentation and pulverization of the electrode, and the active materials may even disconnect from the current collector during cycling. Additionally, solid electrolyte interface (SEI) will repeatedly form on the freshly exposed silicon surface, and the battery capacity decreases sharply with cycling.^[^ ^3^ ^]^

Current studies have improved the comprehensive performance of silicon anode by means of designing specific silicon nanostructure,^[^ ^4^ ^]^ preparation of silicon composite material such as Si/C composite,^[^ ^5^ ^]^ preparation of artificial SEI on the surface of silicon^[^ ^6^ ^]^ and pre‐lithiation of the anodes.^[^ ^7^ ^]^ However, these methods are difficult to be scaled up due to complex processes and high costs. By contrast, high‐performance binders can be directly implemented in the current electrode sheet coating process used by the modern battery industry, which will have great potential for industrialization.^[^ ^8^ ^]^ Polymeric binders have also turned out to play a crucial role in the stabilization of Si electrodes and different types of binders have been reported in recent years, including binders based on hydrogen bond interaction,^[^ ^9^ ^]^ binders containing covalent attachment with Si,^[^ ^10^ ^]^ binders based on ion pair interaction,^[^ ^11^ ^]^ self‐healing binders,^[^ ^12^ ^]^ dynamic crosslinked binders,^[^ ^13^ ^]^ and conductive binders.^[^ ^14^ ^]^ For example, Choi et al. reported a series of topological crosslinking binders based on a molecular pulley to improve the adaptability of volume change during cycling. These binders with specific molecular dynamics were applied to crystalline silicon,^[^ ^15^ ^]^ SiO,^[^ ^16^ ^]^ and Si/C composites^[^ ^17^ ^]^ and showed excellent cycling performance.

The design and application of functional binders can effectively improve the performance of silicon‐based anode. However, there are only a few reports about binders based on ionic polymer conductors with high ionic conductivity. Some recent works prepared ionic conductive binders based on PAA or other carboxylate type polymers by Li‐ion exchange,^[^ ^18^ ^]^ but strong Lewis acid‐base interaction between carboxylate groups and lithium ions limited their conduction capability.^[^ ^19^ ^]^ To further improve the ionic conductivity of the binder to realize outstanding transportation of Li^+^ ions under high‐rate charging/discharging, single‐ion groups such as sulfonimide and lithium borate were introduced in the structure of the binder.^[^ ^20^ ^]^ Qin et al. designed a single‐ion polymer binder for silicon anode based on PEEK containing fluorinated sulfonic side chains and sulfonimide groups, which showed a better rate performance compared to the electrodes using CMC, alginate or PVDF as the binder.^[^ ^21^ ^]^ Zhong et al. reported a slightly crosslinked lithium borate containing single‐ion conducting polymer as the binder for Li‐S batteries, which delivered a high lithium‐ion diffusion coefficient.^[^ ^22^ ^]^ Therefore, a binder with high ionic conductivity will build a continuous conducting network in the electrode for Li‐ion migration, which is expected to reduce the overall resistance, improve ICE, and stabilize the interface of the silicon electrode.

In this work, we propose a slidable and highly ionic conductive flexible polymer binder with a specific single‐ion structure (abbreviated as SSIP) with a dynamic molecular pulley by introducing polyrotaxane as the slidable crosslinker and a sulfimide‐pendent single‐ion oligomer with polyether type main chain (abbreviated as P‐TFMSI‐Li) as the single‐ion conducting segment. Our previous work^[^ ^23^ ^]^ proved that the sulfonimide type segment P‐TFMSI‐Li had strong ion conduction ability, significantly reduced the battery impedance, and contributed to the formation of stable SEI on the electrode surface and the ICE of the electrode is as high as 92%. Owing to the rational design of the SSIP binder, adhesion ability, mechanical properties, and electrochemical properties are taken into account. Slidable polyrotaxnes provide energy dissipation within SSIP and can make the electrode adaptive to the volume change. This loose crosslinked network is formed via molecular sliding during the process of lithiation; when the Li‐Si alloy delithiates, the polymer network shrinks and maintains a stable electrode architecture. Due to the high ionic conductivity and dynamic sliding properties of SSIP, the electrode maintains a high specific capacity after long cycling. In addition, the high‐loading silicon electrodes prepared with SSIP binder achieve high ICE (87.5% ≈ 91.5%) and maintain high areal capacity after cycling.

## 2 Results and Discussion

**Figure 1:**
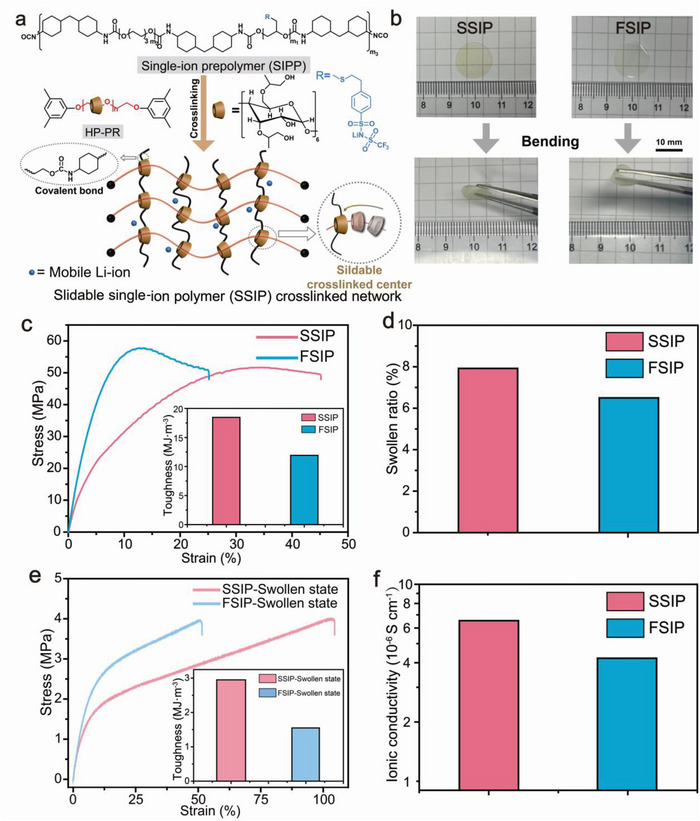
a) Synthesis schematic illustration and segment structure of slidable single‐ion polymer (SSIP) crosslinked network. b) Digital photos of SSIP and FSIP membranes under different states. c) Stress‐strain curves and toughness of SSIP and FSIP membranes at a tensile rate of 1 mm min^−1^. d) Equilibrium swollen ratios (*η*) of SSIP and FSIP membranes. e) Stress‐strain curves and toughness of SSIP and FSIP membranes under swollen state at a tensile rate of 1 mm min^−1^. f) Ionic conductivity of SSIP and FSIP membranes under swollen state.

The synthetic procedure and molecular structure of SSIP and single‐ion polymer binder with a fixed crosslinker (abbreviated as FSIP) are shown in **Figure** **1**a and Figure S3, Supporting Information, respectively. SSIP was crosslinked by hydroxypropyl polyrotaxane (HP‐PR) which contained dynamic crosslinked points, while FSIP was crosslinked by hydroxypropyl cyclodextrin (HP‐CD) with fixed crosslinking junctions. FTIR spectra of SSIP and FSIP are shown in Figure S8, Supporting Information. With the completion of the reaction, the specific absorption peaks of hydroxyl groups in SIPP (Figure S7, Supporting Information) disappeared completely, and significant absorption peaks were assigned to the carbamate group, carbonate group, ether bond, and thionyl group in the polymer chain, which confirmed the molecular structure of the polymer binders. Digital photos of SSIP and FSIP films are shown in Figure 1b. These two polymers appeared to be yellow transparent films, which could be bent and have good flexibility. The tensile properties of SSIP and FSIP are shown in Figure 1c. The yield strengths of SSIP and FSIP were 51.6 MPa and 57.8 Mpa, respectively, and the elongations at breakage were 45% and 25%, respectively. This was mainly attributed to the fact that FSIP had the fixed crosslinker while SSIP crosslinked by HP‐PR had a molecular dynamic pulley‐like effect. Although the mechanical strength of SSIP was slightly lower than that of FSIP, its better deformability preliminarily proved that SSIP had reliable chain mobility, which was helpful to adapt to a high volume change of silicon anode during the charging and discharging cycle. According to Equation (S3) in supporting information, the toughnesses of SSIP and FSIP were 18.5 and 11.9 MJ m^−3^, respectively. The high toughness of the material enabled it to absorb the energy generated during deformation and maintain the overall structure. As shown in Figure 1d, commercial liquid electrolytes were used to swell SSIP and FSIP films. The swelling ratio of SSIP and FSIP were 7.9% and 6.5%, respectively. Generally speaking, if the swelling ratio of the binder is too low, it is difficult for the electrode to be fully wetted by the electrolyte, and the ion transport channel is blocked, resulting in high internal impedance of the battery. However, if the binder is over‐swelled, the mechanical properties will be significantly weakened, and the electrode structure is hard to remain integrity during lithiation and delithiation, leading to the repeated formation of SEI on the surface of silicon, consumption of active lithium ions, and generation of “dead silicon”. Therefore, a moderate swelling ratio is key to the stability of the overall electrode structure during cycling and ensures smooth Li‐ion transport. In order to measure the mechanical properties of polymer binders in the environment of batteries, tensile tests were carried out on swollen SSIP and FSIP films. As shown in Figure 1e, although the breaking strength of both SSIP and FSIP decreased to ≈4 MPa after swelling, the elongation at break increased to 110% and 50%, respectively. With the plasticizing effect of carbonate electrolyte, the dynamics of SSIP with molecular pulley were further boosted. The fracture strain of 110% and the toughness of ≈3 MJ m^−3^ proved that SSIP could adapt to the huge volume change of the silicon anode during cycling and dissipate the energy generated during deformation, which stabilized the electrode structure and improved the overall performance of the battery.

In order to prove that the P‐TFMSI‐Li segment can effectively play the role of ionic conduction in the binder, SSIP and FSIP films were soaked in DEC/EC = 7/3 electrolyte without lithium salts until complete swelling. The ionic conductivity of the films was measured by the electrochemical impedance method and calculated by Equation (S5) in supporting information. Most of the binders did not have the ability to conduct lithium ions, while SSIP and FSIP binders with P‐TFMSI‐Li showed ionic conductivity of 6.5 × 10^−6^ S cm^−1^ and 4.5 × 10^−6^ S cm^−1^ at room temperature, respectively (Figure 1f). Due to the limited swelling ratio of the binder system, the ionic conductivity could not reach the order of 1.0 × 10^−5^ S cm^−1^, but it was still much higher than other nonLi‐ion conductive binders such as CMC, Alg, PAA, and PVDF.^[^ ^9^, ^24^ ^]^ The SSIP and FSIP binders with high ionic conductivity facilitated the conduction of lithium ions inside the electrode and in the interface between the electrode and electrolyte, which were expected to reduce the overall impedance of the battery to achieve better cycling performance.

Next, we prepared electrodes of SSIP:CNT = 1:1 and FSIP:CNT = 1:1 and measured the electrochemical stability of the binders by CV. The reference electrode and counter electrode were lithium metal. As shown in Figure S10, Supporting Information, only weak redox peaks appeared in the first curve in the voltage range of 0.01–1.2 V, and the subsequent four curves were very stable and almost overlapped with each other. Such observation proved that single‐ion polymer binders possessed excellent electrochemical stability in the working voltage range of silicon anode. As shown in Figure S11, Supporting Information, we tested the thermal stability of the binder through thermogravimetric experiments. SSIP and FSIP only lost 10% of the mass under 300 °C. In addition, the thermogravimetric curve of the Si@SSIP electrode was similar to that of SSIP. The active material silicon powder and conductive carbon nanotubes in the electrode were stable under 800 °C in a nitrogen atmosphere. About 20% of the mass loss in the test came from the decomposition of SSIP, corresponding to the binder ratio in the electrode.

**Figure 2:**
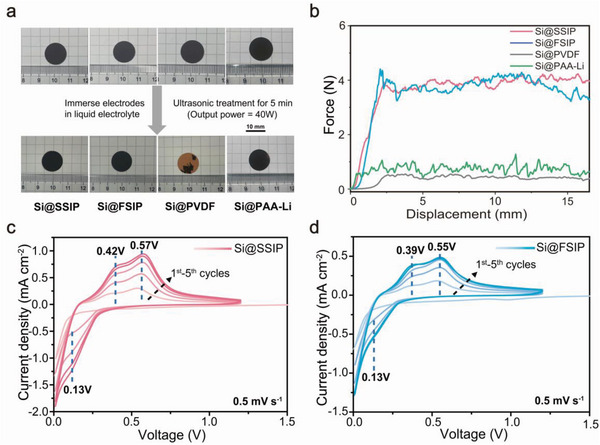
Performance of electrodes based on different binders. a) Digital photos of Si@SSIP, Si@FSIP, Si@PVDF, and Si@PAA‐Li under dry state and digital photos of these electrodes after ultrasonic treatment under swollen state. b) Peeling force‐displacement curves of Si@SSIP, Si@FSIP, Si@PVDF and Si@PAA‐Li. c) CV curves of Si@SSIP at a scan rate of 0.5 mV s^−1^. d) CV curves of Si@FSIP at a scan rate of 0.5 mV s^−1^.

Digital photos of prepared electrodes are shown in **Figure** **2**a. As the silicon powder and CNT in all electrodes were in the same proportion, the appearance of the electrodes was almost the same. The electrodes were soaked in the electrolyte until fully swollen and then treated with ultrasound for 5 min. The macroscopic morphologies of Si@SSIP and Si@FSIP electrodes were nearly unchanged after ultrasonic treatment, while most of the active substances on the surface of the Si@PVDF electrode fell off after ultrasonication, exposing more than 75% area of the copper foil. Also, a small part of the anode materials separated from Si@PAA‐Li after ultrasonic treatment. These experiments clearly showed the stronger adhesion of SSIP and FSIP binders compared to that of traditional binders, especially PVDF.

The adhesion performances of crosslinked binders of SSIP and FSIP were further proved quantitatively by the peeling test. As shown in Figure 2b, Si@SSIP and Si@FSIP exhibited similar peeling force (≈4 N), while the peeling force of Si@PVDF and Si@ PAA‐Li were 0.5N and 1N, respectively. Abundant carbamate groups in SSIP and FSIP formed a large number of hydrogen bonds in polymer‐polymer and polymer‐silicon, inducing strong noncovalent interaction inside the electrode. In addition, the high crosslinking density in the system created a complete polymeric network, and the cohesion forces of SSIP and FSIP were much higher than that of PVDF and PAA‐Li with linear structure due to the high molecular weight and entanglement of crosslinked polymer.

The SEM images of Si@SSIP, Si@FSIP, and Si@PVDF electrodes before cycling were shown in Figure S12, Supporting Information. The conductive agent CNT was evenly distributed in the electrode to form a complete conductive network, and the ionic conductive binder provided a continuous Li‐ion transfer channel for the electrode. The electrode structure before cycling showed no cracks or defects.

The electrochemical properties of Si@SSIP and Si@FSIP electrodes were characterized by CV with lithium metal as reference electrode and counter electrode. As shown in Figure 2c, the lithium reduction peak at 0.01 V could be found in the first cathodic scan, which indicated the formation of an amorphous Li_x_Si_y_ alloy by the electrochemical reaction between crystalline silicon and lithium ions. In subsequent cathodic scans, a new reduction peak appeared at 0.13 V, which was assigned to the reversible conversion between amorphous silicon and Li_x_Si_y_ alloy. At 0.42 and 0.57 V, two oxidation peaks appeared, which were assigned to the delithiation process. With the increase of scanning number, the intensity of the redox peaks also increased gradually, indicating that more crystalline silicon was activated to participate in the electrochemical reaction. The Si@FSIP electrode also showed a similar redox process (Figure 2d), consistent with the typical CV curve of lithiation/delithiation characteristics of crystalline silicon electrodes.

**Figure 3:**
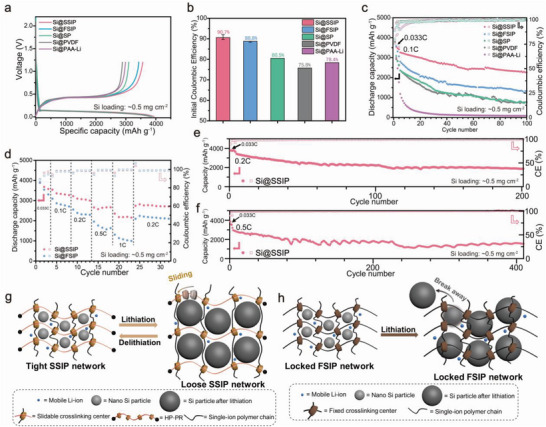
a) First charging and discharging curves of Si electrodes with various binders at 0.033C. b) ICE of Si electrodes with various binders at 0.033C. c) Cycling performance of Si electrodes with various binders at 0.1C. d) Rate performance of Si@SSIP and Si@FSIP electrodes. e) Cycling performance of Si@SSIP electrode at 0.2C. f) Cycling performance of Si@SSIP electrode at 0.5C. g) Schematic illustration for structure change of Si@SSIP electrodes during charging and discharging. h) Schematic illustration for structure change of Si@FSIP electrodes during charging and discharging. Si loading of the electrodes was ∼0.5 mg cm^−2^.

To better demonstrate the comprehensive battery performance of the silicon electrode, we first activated the electrode with a small current. **Figure** **3**a showed the first charge–discharge curves of Si@SSIP, Si@FSIP, Si@SP, Si@PVDF and Si@PAA‐Li electrodes at 0.033C (1C = 4200 mA g^−1^). The corresponding initial Coulombic efficiency (ICE) was calculated in Figure 3b. Error bars were derived from the determination of multiple batteries with the same batch of electrodes. The ICEs of Si@SSIP and Si@FSIP electrodes were 90.7% (highest up to 92.0%) and 88.8%, respectively, while the ICEs of Si@FSIP, Si@SP, Si@PVDF, and Si@PAA‐Li electrodes were 80.5%, 75.8%, and 78.4%, respectively. The introduction of lithium sulfonimide conductive binder improved interface compatibility between electrode and electrolyte, and at the same time, the formation of stable SEI only required a small number of active lithium ions. ICEs of SSIP and FSIP were significantly higher than those of the electrodes prepared by nonconductive binders.

After three cycles of activation at 0.033C, the cycling performances of silicon electrodes with different binders at 0.1C are shown in Figure 3c. After 100 cycles, the discharge‐specific capacities of Si@SP and Si@PVDF were 760 and 739 mAh g^−1^, respectively, while that of Si@FSIP with single‐ion binder was 1334 mAh g^−1^. The introduction of a molecular pulley in the binder significantly improved the capacity retention rate of the electrode, which could provide a high discharge capacity of 2264 mAh g^−1^ even after 100 cycles. In order to eliminate the influence of a small amount of thickener PAA‐Li in the system on the battery performance, the Si@PAA‐Li electrode was prepared separately. After complete lithiation, the strong hydrogen bond between the segment of PAA was destroyed, and the overall performance decreased significantly. After 25 cycles, the electrode capacity almost decayed to 0. It was proved that the introduction of a small amount of PAA‐Li only played a thickening role and did not have a positive impact on the electrochemical performance of the electrode. CNT is more suitable for silicon anode because of its one‐dimensional conductivity compared with SP. To further confirm the high ICE of the electrode was independent of CNT, Si@SSIP‐SP and Si@PVDF‐SP were also prepared with traditional conductive agent SP. As shown in Figure S13a—c, Supporting Information, the ICE of Si@SSIP‐SP was 85.48%, while that of Si@PVDF‐SP was only 62.32%, which suggested the electrode with our synthesized slidable and highly ionic conductive flexible polymer binder still had higher ICE when using SP as the conductive material. We also compared the cycling performance of Si@SSIP‐SP and Si@PVDF‐SP for the first ten cycles in Figure S13d, Supporting Information, and it suggested that Si@SSIP‐SP had more excellent cycling capability.

As shown in Figure S14, Supporting Information, we conducted EIS on Li||Si half cells to measure the impedance. Si@SSIP, Si@FSIP, Si@SP, and Si@PVDF were used as working electrodes, and lithium metal was used as the counter electrode and reference electrode. The data are recorded in Table S2, Supporting Information and an equivalent circuit diagram is shown in Figure S13e, Supporting Information, where *R*_s_, *R*_SEI_, *R*_ct_, W, and CPE represent electrolyte impedance, SEI film impedance, charge transfer impedance, Warburg impedance, and constant phase element, respectively.

The electrodes using single‐ion binders displayed lower charge transfer impedance. The *R*_ct_ of Si@SSIP and Si@FSIP were 33 and 65 Ω, respectively. The *R*_ct_s of Si@PVDF and Si@SP were 545 and 188 Ω, respectively. In the case of similar electronic conductivity, the excellent ionic conductivity of SSIP and FSIP binders significantly reduced the interface impedance between electrolyte and electrode. The battery with smaller internal resistance was beneficial to achieving better performance.

On the one hand, after 100 cycles, the *R*_ct_s of Si@SSIP and Si@FSIP decreased significantly, mainly because the active substances of the electrode were fully infiltrated and activated after long‐term charging and discharging. For comparison, after cycling, lots of particles in Si@PVDF and Si@SP became inactive, which hindered the charge transfer and resulted in a high *R*_ct_. On the other hand, a silicon electrode with single‐ion binders also exhibited a lower *R*_SEI_, proving that an ionic conductive network effectively promoted ion conduction in the solid/liquid and electrode/electrolyte interface.

The surface morphology of electrodes after cycling was observed by SEM. As shown in Figure S15, Supporting Information, a large number of cracks appeared on the surface of Si@PVDF and Si@FSIP electrodes after cycling, and the electrodes showed an obvious trend of pulverization. However, only a small amount of shallow depth cracks showed up in the Si@SSIP electrode. This was mainly because of the excellent dynamic characteristics of SSIP binder, which made it adaptive to the expansion and contraction during cycling, thus showing outstanding cycling stability.

The content and valence states of elements on the electrode before and after cycling were characterized by XPS. As shown in Figure S16, Supporting Information, after cycling, the F contents of Si@SSIP and Si@FSIP were 3.14% and 3.40%, respectively, which was much higher than the 1.75% of Si@PVDF. Although the PVDF polymer itself has the highest F content (Figure S15b, Supporting Information). LiF is currently recognized as the most stable inorganic SEI component.^[^ ^25^ ^]^ The F content preliminary proved that a single‐ion binder induced an SEI with a higher amount of LiF. Figure S17, Supporting Information shows full XPS spectra and high‐resolution XPS spectra with related peaks fitting results of C 1s, F 1s, and Si 2p of Si@SSIP, Si@FSIP, and Si@PVDF before cycling. The four signal peaks fitted in the spectra of C 1s were located at ≈290.1, ≈288.1 eV, ≈286.1, and ≈283.8 eV, which were assigned to C–F, C=O, C—O, and C—C, respectively, corresponding to the molecular structure of the binders. The two signal peaks fitted in Si 2p spectra were located at ≈102.5 and ≈98.5 eV, which were assigned to Si‐O and crystalline silicon. This was mainly because the atoms on the surface of crystalline silicon oxidized spontaneously in the air to form SiO_2_. SiO_2_ on the surface of silicon particles had abundant hydrogen bond receptors and formed strong interactions with binders based on polyurethane systems.

Figure S18, Supporting Information shows full XPS spectra and high‐resolution XPS spectra with related peaks fitting results of C 1s, F 1s, and Si 2p of Si@SSIP, Si@FSIP, and Si@PVDF after cycling. A complete and compact SEI was formed on the surface of the electrodes after cycling, and the components of SEI could be well analyzed by XPS. The three peaks fitted in the C 1s spectra were located at ≈290.0, ≈286.5, and ≈284.0 eV, assigned to C=O, C—O, and C–C, respectively. The peak attributed to C=O in Si@PVDF was the highest, which proved that the SEI component had a high content of Li_2_CO_3_. The three peaks of Li 1s spectra were located at ≈55.8, ≈54.8, and ≈54.0 eV, which were assigned to LiF, Li_2_CO_3_, and ROCO_2_Li, respectively. The contents of Li‐F in the SEI of Si@SSIP and Si@FSIP were higher, while the main SEI component of Si@PVDF was Li_2_CO_3_. The three signal peaks fitted in the F 1s spectra were located at ≈688.0 and ≈684.5 eV, which were assigned to C–F and Li‐F, respectively, corresponding to the organic C–F component and inorganic LiF component in SEI.

Rate performance is a very important evaluation parameter for silicon electrodes, which measures the comprehensive performance of the battery under the condition of fast charging and discharging. As shown in Figure 3d, we evaluated the rate performances of Si@SSIP and Si@FSIP electrodes. The specific capacity of Si@SSIP electrode at current densities of 0.1, 0.2, 0.5, and 1C was 3331, 3069, 2643, and 2182 mAh g^−1^, respectively. When the current density returned to 0.2C, the specific capacity of the electrode quickly recovered to 2816 mAh g^−1^, proving that the structure of the electrode was reversible after undergoing a higher current density. The specific capacities of Si@FSIP electrodes were 2807 and 2310 mAh g^−1^ at current densities of 0.1 and 0.2C, respectively. However, the capacity of the electrode dropped rapidly at higher current densities. The discharge capacity of the electrode at 0.5C was 1654 mAh g^−1^, and when the current density increased to 1C, the specific capacity of Si@FSIP was only 1078 mAh g^−1^. The results showed that Si@FSIP electrodes with fixed crosslinking centers performed poorly under a high current density.

As shown in Figure 3e,f, we measured the long‐cycle performance of the Si@SSIP electrode at current densities of 0.2 and 0.5C, after three activation cycles with a current density of 0.033C. At a current density of 0.2C, the electrode provided a discharge capacity of 1867 mAh g^−1^ after 200 cycles, and the average CE was 99.31% from the 5th to the 200th cycle. At a current density of 0.5C, the electrode delivered a discharge capacity of 1620 mAh g^−1^ after 400 cycles with a high average CE of 99.63% from the 5th to the 400th cycle.

Based on the result of the rate test, Si@SSIP demonstrated better performance at high current density. It also showed excellent long‐cycle performance at different current densities. The schematic diagram of the structure change of the Si@SSIP electrode during charging and discharging is shown in Figure 3g. In the process of lithiation of silicon, the molecular pulley structure of the SSIP binder was able to dynamically adjust to the volume change, and the dynamic crosslinking network remained stable during charging and discharging. The schematic diagram of the structure change of the Si@FSIP electrode during charging and discharging is shown in Figure 3h. The Si@FSIP electrode without dynamic bonds was difficult to adapt to the change of electrode volume during cycling or undergoing a high current density.

**Figure 4:**
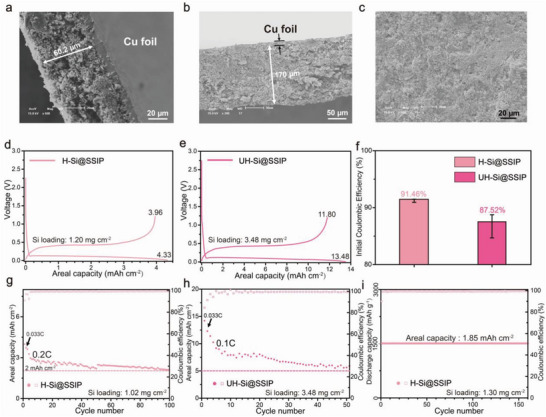
a) Cross‐section SEM image of H‐Si@SSIP electrode. b) Cross‐section SEM image of UH‐Si@SSIP electrode. c) Top‐view SEM image of UH‐Si@SSIP electrode. d) First charging and discharging curves of H‐Si@ SSIP electrode with a Si loading of 1.20 mg cm^−2^ at 0.033C. e) First charging and discharging curves of UH‐Si@SSIP electrode with a Si loading of 3.84 mg cm^−2^ at 0.033C. f) ICE of H‐Si@SSIP and UH‐Si@SSIP electrodes. g) Cycling performance of H‐Si@SSIP electrode at 0.2C. Si loading = 1.02 mg cm^−2^. h) Cycling performance of UH‐Si@SSIP electrode at 0.1C. Si loading = 3.84 mg cm^−2^. i) Cycling performance of H‐Si@SSIP electrode with limited discharge capacity of 1500 mAh g^−1^ (Areal capacity = 1.85 mAh cm^−2^) at 0.15C. Si loading = 1.30 mg cm^−2^.

To further prove the practicality of SSIP, a high areal capacity silicon anode (H‐Si@SSIP) with an active loading of 1.25 ± 0.35 mg cm^−2^ and an ultra‐high areal capacity silicon anode (UH‐Si@SSIP) with an active loading of 3.50 ± 0.55 mg cm^−2^ were prepared. As shown in **Figure** **4**a,b, the thickness of the H‐Si@SSIP electrode was 60.2 µm, and that of the UH‐Si@SSIP electrode reached 170 µm. As shown in Figure 4c, we found that even in the case of ultra‐high silicon loading, the surface morphology of the electrode was essentially uniform and complete, and no obvious structural defects, such as cracks existed before cycling.

We used a small current density of 0.033C to activate the electrode and measure the charge and discharge capacity at the first cycle and ICE of the electrode. The error bars came from the measurement of multiple batteries with the same batch of electrodes. As shown in Figure 4d, when the silicon loading increased from ≈0.5 mg cm^−2^ to 1.25 ± 0.35 mg cm^−2^, the ICE of the electrode hardly changed and was still above 90%. The areal discharge capacity of the first cycle was 4.33 mAh cm^−2^ and the areal charging capacity of the first cycle was 3.96 mAh cm^−2^ with a high ICE of 91.46%. When the silicon loading further increased, the ICE of the electrode slightly decreased, which might be related to the increase of specific surface area, and the volume of the electrode was greatly increased. The areal discharge capacity of the first cycle of the UH‐Si@SSIP electrode with a silicon loading of 3.84 mg cm^−2^ was 13.48 mAh cm^−2^, and the areal charging capacity was 11.80 mAh cm^−2^ with an ICE of 87.52% (Figure 4e,f).

As shown in Figure 4g, we tested the long‐term stability of the H‐Si@SSIP electrode at a current density of 0.2C. After 100 cycles, the discharge areal capacity of 2.10 mAh cm^−2^ was maintained. When the capacity of the electrode was set to constant 1500 mAh g^−1^ (corresponding areal capacity was 1.85 mAh cm^−2^), the electrode could steadily charge/discharge for more than 160 cycles at a current density of 0.15C (Figure 4i). As shown in Figure 4 h, after 3 cycles of activation at 0.033C, we conducted cycling test on the UH‐Si@SSIP electrode with a high silicon loading of 3.84 mg cm^−2^. After 50 cycles, the areal discharge capacity of 5.90 mAh cm^−2^ of the anode was displayed, which was much higher than the areal capacity level required for most commercial electrodes (3 mAh cm^−2^). Also, we set the specific capacity of the UH‐Si@SSIP electrode with a silicon loading of 3.43 mg cm^−2^ to fixed 1500 mAh g^−1^ (corresponding areal capacity of 5.15 mAh cm^−2^), and the electrode could stable charge/discharge for more than 70 cycles at 0.1C, which further proved the commercial application prospect of this binder (Figure S19, Supporting Information). The high areal capacity silicon electrode demonstrated its high ICE and reliable cycle stability, which proved that the design strategies of introducing single‐ion conductive groups and molecular pulley into the structure of binder structure were effective in improving the performance of the silicon anode.

**Figure 5:**
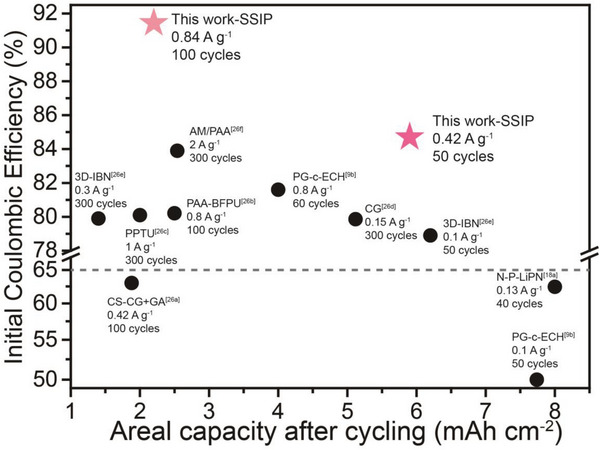
Areal capacity after cycling and relevant ICE of Si electrodes based on different binders from references^[^ ^9^, ^18^, ^26^ ^]^ and SSIP.

Although research has been carried out to design functional binders for silicon anodes, most of the high areal capacity silicon anodes had low ICE, resulting in a huge loss of active Li‐ion in the first few cycles, which was not suitable for practical application. The electrode based on SSIP had a record high areal capacity with a reliable ICE (**Figure** **5**).

## 3 Conclusion

A polymer binder SSIP for high‐performance Si anodes was prepared via rational structure design. By introducing ionic conductive oligomer P‐TFMSI‐Li into the binder, the ionic conductivity of the binder was remarkably improved. The overall impedance of the battery was greatly reduced and more stable SEI was induced on the surface of the silicon electrode resulting in better stability. Also, the ICE of the electrode with SSIP was significantly improved (up to 92.0%). By introducing a dynamic molecular pulley in the binder structure via crosslinking the polymer by PR, the cycle stability and rate performance of the silicon anode were greatly improved. Si@SSIP provided a discharge capacity of 2264 mAh g^−1^ after 100 cycles at 0.1C, which was significantly higher than electrodes with other binders. Si@SSIP also delivered a specific capacity of 1867 mAh g^−1^ after 200 cycles at 0.2C and 1620 mAh g^−1^ after 400 cycles at 0.5C with a high average coulombic efficiency. In order to prove the practicability of the novel binder, we prepared high areal capacity silicon electrode H‐Si@SSIP and ultra‐high areal capacity silicon negative electrode UH‐Si@SSIP. H‐Si@SSIP and UH‐Si@SSIP showed a high ICE of 91.5% and 87.5%, respectively, which effectively improved the efficiency of the active material of the negative silicon electrode. The high areal capacity electrode still displayed desirable cycle stability, providing the areal capacity of 2.1 mAh cm^−2^ after 100 cycles of H‐Si@SSIP and 5.90 mAh cm^−2^ after 50 cycles of UH‐SSIP. Silicon anode prepared by SSIP is expected to be applied to lithium‐ion batteries to achieve a higher energy density.

## Conflict of Interest

The authors declare no conflict of interest.

## Supporting information

Supporting Information

## Data Availability

The data that support the findings of this study are available from the corresponding author upon reasonable request.
