# Supplementary material for: Slidable and Highly Ionic Conductive Polymer Binder for High‐Performance Si Anodes in Lithium‐Ion Batteries
Source: Adv Sci (Weinh). 2022 Dec 23;10(6):2205590. doi: 10.1002/advs.202205590 (PMC9951352; doi:10.1002/advs.202205590)
Supplement: Supplementary file 1 — Supporting Information [file ADVS-10-2205590-s001.pdf]

ADVANCED SCIENCE
Open Access

Supporting Information

for *Adv. Sci.*, DOI 10.1002/advs.202205590

Slidable and Highly Ionic Conductive Polymer Binder for High-Performance Si Anodes in Lithium-Ion Batteries

Yifeng Cai, Caixia Liu, Zhiao Yu, Wencan Ma, Qi Jin, Ruichun Du, Bingyun Qian, Xinxin Jin, Haomin Wu, Qiuhong Zhang and Xudong Jia**

Supporting Information

Slidable and Highly Ionic Conductive Polymer Binder for High Performance Si Anodes in Lithium-Ion Batteries

A Highly-Adhesive and Self-healing Elastomer for Bio-Interfacial Electrode

Yifeng Cai, Caixia Liu, Zhiao Yu, Wencan Ma, Qi Jin, Ruichun Du, Bingyun Qian, Xinxin Jin, Haomin Wu, Qiuhong Zhang and Xudong Jia**

Materials

4,4′-Diisocyanate dicyclohexylmethane (HMDI, 90%, Sigma-Aldrich), polycarbonate diol (PCDL1000/PCDL1k, *Mn*=1000, UBE Corporation), dibutyltin dilaurate (DBTL, 95%, Sigma-Aldrich), polyethylene glycol (PEG4000/PEG4k, *Mn*=4000, Macklin) were dried under vacuum at 85 °C overnight before use. N,N-Dimethylformamide (DMF, AR, General-Reagent) was dried by CaH_2 and vacuum transferred. Tetrahydrofuran (THF, AR, Macklin) was dried by CaH_2 and distilled. p-Toluenesulfonyl chloride (p-TS, >99%, TCI), α-cyclodextrin (α-CD, HPLC, Macklin), 2-hydroxypropyl-β-cyclodextrin (HP-CD, ≥99.5%, Macklin), 3,5-dimethylphenol (98%, Alfa Aesar), propylene oxide (PO, 99%, Macklin), polyacrylic acid (PAA, *Mw*=450 kDa, Macklin), lithium carbonate (Li_2CO_3, 99.5%, Macklin), nano silicon powder (nano-Si, APS≤50 nm 98%, Macklin), diethyl carbonate (DEC, 99%, Macklin), ethylene carbonate (EC, HPLC, Macklin), polyvinylidene fluoride (PVDF, Arkema), commercial liquid electrolyte (1M LiTFSI in DEC/EC=1/1 V/V with 10% FEC & 1% VC, DoDo Chem), Super P conductive carbon black (SP, Timcal), dobby carbon nanotubes (CNT, carboxylic acid functionalization, inner diameter = 5-10 nm, outer diameter = 10-20 nm, length = 10-30 μm, J&K Scientific) and Li metal foil (Battery grade, China Energy Lithium co., Ltd) were used without further purification. The separator Celgard 2500 was supplied from Celgard LLC.

Synthesis

Synthesis of *P-TFMSI-Li*

The detailed synthesis and structural characterization of **P-TFMSI-Li** were reported by our previous work.[1]

Synthesis of *HP-PR*

Figure S1 shows the synthesis route for hydroxypropyl polyrotaxane (HP-PR). The preparation of HP-PR required a multi-step reaction starting from PEG4k. The detialed synthesis route referred to our previous work.[2]

Synthesis of ***PEG-Ts***.

8 g PEG4k (2.0 mmol) was placed in a vacuum oven at 85 °C overnight to remove trace water. Under the protection of argon, 30 ml of anhydrous THF was added to dissolve PEG in a 250 ml three-necked flask. After that, 1.6 ml TEA (11.5 mmol) and 2.86 g p-toluenesulfonyl chloride (15.0 mmol) which were dissolved in 10 ml of anhydrous THF were added and the mixture was stirred at room temperature for 24 h. After the reaction finished, THF was removed by rotary evaporator. The crude product was dissolved in 30 ml DCM and poured into 500 ml diethyl ether for precipitation. The purified product was collected by filtration and dried at 85 °C to obtain PEG-Ts (7.1 g, 81.8%). The ^{1}H-NMR spectrum of PEG-Ts is shown in **Figure S4**. The peaks appeared at 7.82-7.25 ppm corresponded to the benzene and the peaks appeared at 2.40-2.30 ppm corresponded to the methyl. The board peak located at 3.60 ppm was the characteristic peak of methylene in PEG4k. The integral ratio of the three kinds of hydrogen was 8:371:6, which was close to the theoretical value.

Synthesis of ***Pre-PR***.

7.1 g PEG-Ts was dissolved in 160 ml deionized water. 31.6 g α-CD (32.5 mmol) was dissolved in 240 ml deionized water. The two kinds of solution were mixed and further sonicated for 1 h. The self-assembly was completed by mechanical stirring for 24 h at room temperature. Water was removed by rotary evaporator. The product was fully dried in a vacuum oven at 85 °C for 36 h to obtain Pre-PR, which was further ground into powder in a mortar.

Synthesis of ***PR***.

8.6 g 3,5-dimethylphenol (7.0 mmol) was dissolved in 60 ml anhydrous DMF in a 250 ml three-necked flask, 4.5 g NaH (112.5 mmol) was added slowly in an ice bath and the mixture was stirred for 0.5 h. 10 g Pre-PR powder was then added slowly with intensely stirring and another 15 ml anhydrous DMF was added for dilution. After stirring well, the system was transferred to 30 °C and continued to react for 24 h. The crude product was poured into 1200 ml methanol for precipitating and washing, the precipitate was washed twice with 1200 ml and 600 ml methanol, respectively. The precipitate was purified by centrifugation to remove methanol. The centrifugal product was mixed solution with 30 ml DMSO and 30 ml deionized water was added to form a dispersion, which was dialyzed for 72 h with changing water every 8 h. After dialysis, water was removed by rotary evaporator and the product was dried under

vacuum at 85 °C for 24 h to obtain 2.24 g PR. The ringing rate was calculated by integration of the NMR spectrum.

The ^{1}H-NMR spectrum of PR is shown in **Figure S5**. After self-assembly and end-capping reactions, the characteristic peaks of the two kinds of end-group benzene ring hydrogen appeared at 6.65 ppm and 6.56 ppm, respectively. The characteristic peak of phenylmethyl located at 2.23 ppm. The characteristic peak of hydrogen on C_1 at α-CD located at 4.76 ppm. The peak at 3.6 ppm was the characteristic peak of methylene in PEG4k and the area integral was 4.23. The number of rings can be calculated by the following formula:

$$\text{Number of α-CD on PEG} = \frac{H_{\text{CD-}C_1} / 6}{H_{\text{PEG}} / 360} \quad (1)$$

In the formula, $H_{\text{CD-}C_1}$ is the value of the integration area of hydrogen on C_1 at α-CD, and H_{PEG} is the value of the integration area of hydrogen on the PEG main chain. It can be calculated that the number of α-CD on each PEG chain was 14.

*Synthesis of **HP-PR**.*

2.24 g PR (~41.5 mmol -OH) was dissolved in 100 ml 8 wt% NaOH solution in a 250 ml three-necked flask. The reaction system was transferred in an ice bath and 42.8 g of epoxypropane (738 mmol) was added dropwise for 4 h. After that, the temperature was recovered to 25 °C and the reaction continued for 24 h. Then the solution was dialyzed for 48 h with changing water every 8 h. After dialysis, water was removed by rotary evaporator and the product was dried under vacuum at 85 °C for 24 h to yield HP-PR 2.10 g. The substitution rate was calculated by integration of the NMR spectrum.

The ^{1}H-NMR spectrum of HP-PR is shown in **Figure S6**a. The peak appeared at 5.10-4.76 ppm belonged to hydrogen on C_1 at α-CD. The peaks around 1.0 ppm corresponded to methyl on hydroxypropyl group. The modifier rate can be calculated by the following formula:

$$\text{Modification ratio} = \frac{H_{\text{HP-CH}_3} / 3}{H_{\text{CD-C}_1}} \quad (2)$$

In the formula, $H_{\text{HP-CH}_3}$ is the value of the integral area of methyl characteristic peak, and $H_{\text{CD-C}_1}$ is the value of integral area of hydrogen on C_1 at α-CD. The modification number was calculated to be 1.93 and the substitution rate was 64.3%. The FT-IR spectrum of HP-PR is shown in Figure S6b. The prepared HP-PR had good solubility in DMF.

Synthesis of single-ion polymer binders:

*Synthesis of single-ion prepolymer (**SIPP**).*

Before reaction, P-TFMSI-Li, HMDI, PCDL1k, HP-PR and HP-CD were placed in a vacuum oven at 85 °C overnight to remove trace water. DMF was dried with CaH_2 and distilled to remove water to avoid the effect of trace moisture in the reactants and solvents on the polymerization. The synthesis steps of the single ionic prepolymer are shown in **Figure S2**. In the formula m_1 equaled 18 and m_2 was 6~7. Under the protection of argon, 250 mg PCDL1k, 394 mg HMDI and 3 mg DBTL were added to a three-necked flask and the mixture was stirred at 85 °C for 2.5 h. During the reaction, 1 ml DMF was added for dilution. Then, P-TFMSI-Li (20 wt% in DMF) was added and reacted for 2 h, and 2 ml DMF was added for dilution. The single-ion prepolymer (SIPP), stored in solution, was obtained.

The ^{1}H-NMR spectrum and FT-IR spectrum of SIPP is shown in **Figure S7**. In the spectrums, all characteristic peaks from different segments were appeared, which verified the structure of SIPP. A large amount of residual NCO in the prepolymer ensured its high activity in the subsequent reaction.

Synthesis of polymer binders.

The synthesis routes for SSIP and FSIP are shown in **Figure 1** and **Figure S3**. After adding HP-PR or HP-CD which served as chain extender to the SIPP, the solution reacted at 85 °C for another 2 h. 8 ml DMF was added to dilute and controlled the viscosity of the solution. After that, the solution was cooled to room temperature and diluted by 10 ml DMF to obtain the polymer solutions of SSIP and FSIP. For comparison, we prepared polymer binder without P-TFMSI-Li, named slidable polymer (SP). The formulas of the polymer binders are shown in the **Table S1**. The polymer binder solutions were stored in a refrigerator at 4 °C. The polymer films of SSIP and FSIP were prepared by pouring the solution in a round PTFE mould to dry the solvent and finish crosslinking reaction in an oven at 85 °C.

Preparation of the Si anodes

To prepare uniform slurries, a small amount of high molecular weight polymer PAA, which was further lithiated to PAA-Li by Li_2CO_3, was added to the SSIP, FSIP and SP solutions and the mass ratio of polymer to PAA-Li was 3:1. Subsequently, the slurry was prepared according to the mass ratio of Si:Binder:CNT=7:2:1. And the slurry was evenly distributed on the copper foil with a scraper, and the silicon electrodes were obtained after vacuum drying at 85 °C overnight. The silicon electrodes prepared with different binders were named as Si@SSIP, Si@FSIP and Si@SP, respectively. And we prepared slurries with Si:PVDF:CNT=7:2:1 and Si:PAA-Li:CNT=7:2:1. The solvents used in the preparation of Si@PVDF and Si@PAA-Li were NMP and DMF, respectively. The silicon electrodes were

prepared in the same way and named as Si@PVDF and Si@PAA-Li. For comparison, we also prepared slurries with Si: SSIP:SP=7:2:1 and Si: PVDF:SP=7:2:1 and named as Si@SSIP-SP and Si@PVDF-SP for electrodes preparation.

Assembly of Li||Si half cells

To assemble Li||Si half cells, the as-prepared electrodes were assembled into 2032 coin-type cells in a glove box (Mikrouna, H_2O and O_2 < 0.01 ppm). The Li foil was used as the counter electrode and reference electrode and Celgard 2500 was used as the separator. 1.0 mol L^{-1} Lithium hexafluorophosphate ($LiPF_6$) in a binary solvent of diethyl carbonate (DEC) and ethylene carbonate (EC) (1:1 in volume) containing 10 vol% fluoroethylene carbonate (FEC) and 1 vol % vinylene carbonate (VC) was used as electrolyte. During cycling, specific capacity and current density were only based on the mass of Si on the electrode.

Material characterization

^{1}H NMR spectra were recorded on a Bruker AVANCE 400MHz III spectrometer using deuterated reagent as solvent with TMS (internal reference). Infrared radiation spectra were collected using Nicolet iS10 infrared spectromete with the wavenumber range of 4000 cm^{-1} ~ 400 cm^{-1}. Mechanical tensile-stress experiment of polymer films and peel test of electrodes were performed on Instron 5944. Polymer films were cut into 2 mm × 35 mm bar samples and the tension rate was set to 1 mm min^{-1}.

Toughness during tensile-stress tests was calculated using equation (3):

$$\text{Toughness} = \int_0^{\varepsilon} f(\varepsilon) d\varepsilon_{stretch\ process} \qquad (3)$$

Where ε represents strain and f(ε) is the function of stress-strain curve in stretch process.

The solvent uptake experiment was conducted by soaking a piece of dry membrane in liquid electrolyte. The solvent uptake ratio was calculated using equation (4):

$$Solvent\ uptake\ ratio(\eta) = \frac{w_t - w_0}{w_0} \times 100\% \qquad (4)$$

Where w_0 is the initial weight of the dry membrane and w_t is the weight of the wet membrane. The weight of the swollen membrane was recorded until equilibrium. Thermogravimetric analysis (TGA) was performed under a nitrogen gas atmosphere on ASAP2020 device from NETZSCH (Germany) with a heating rate of 10 °C min^{-1} from room temperature to 800 °C. Scanning electron microscopy (SEM) was used to investigate the morphology of the Si anodes by S-3400N Hitachi (Japan). X-ray photoelectron spectroscopy (XPS) spectra were measured by a PHI-5000versaprobeIII system with an Al Kα radiation (1486.6 eV) source.

Electrochemical measurements

Electrochemical measurements of polymer membranes were conducted on an electrochemical workstation (CHI660E). The ion conductivity of the polymer membranes was measured with electrochemical impedance spectroscopy (EIS) conducted from 100 kHz to 1 Hz with the voltage amplitude of 10 mV. A symmetrical coin cell prepared by sandwiching the polymer membrane between two stainless steel foils was assembled for test. Before cells packaging, polymer films were prepared by soaked up with DEC/EC=1/1 (V/V).

Ionic conductivity of the electrolyte was calculated using the following equation:

$$\sigma = \frac{1}{R} \cdot \frac{l}{S} \quad (5)$$

in which l refers to the thickness of electrolyte, S is the area of electrolyte, and R is the ohmic resistance of electrolyte.

To evaluate battery performance, charge-discharge tests were carried out by LAND battery test system between 0.01 V and 1.2 V. CV measurement of the electrode was recorded on an electrochemical work station between 0.01 V and 1.2 V at a current density of 0.5 mV s^{-1}. EIS of the electrode was recorded by an electrochemical work station with amplitude of 10 mV in the frequency range of 100 kHz-0.01 Hz.

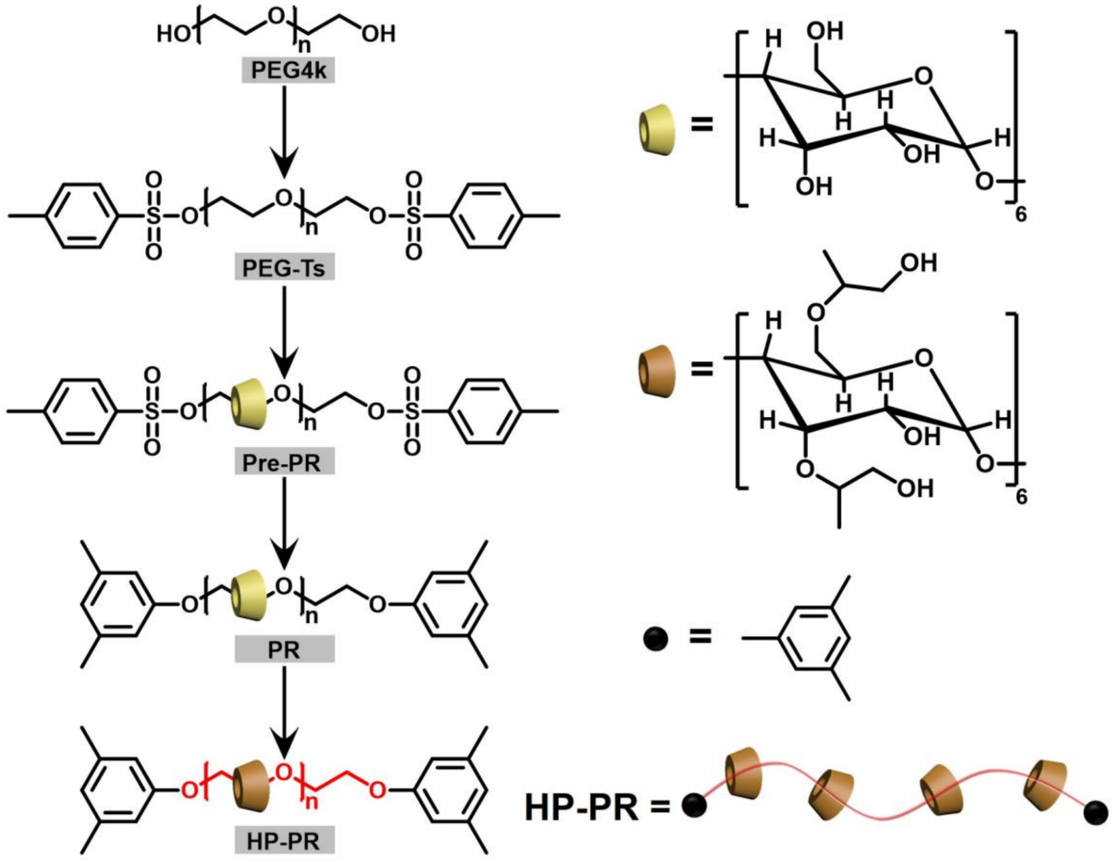

Figure S1. Synthesis schematic illustration of hydroxypropylation-polyrotaxane (HP-PR).

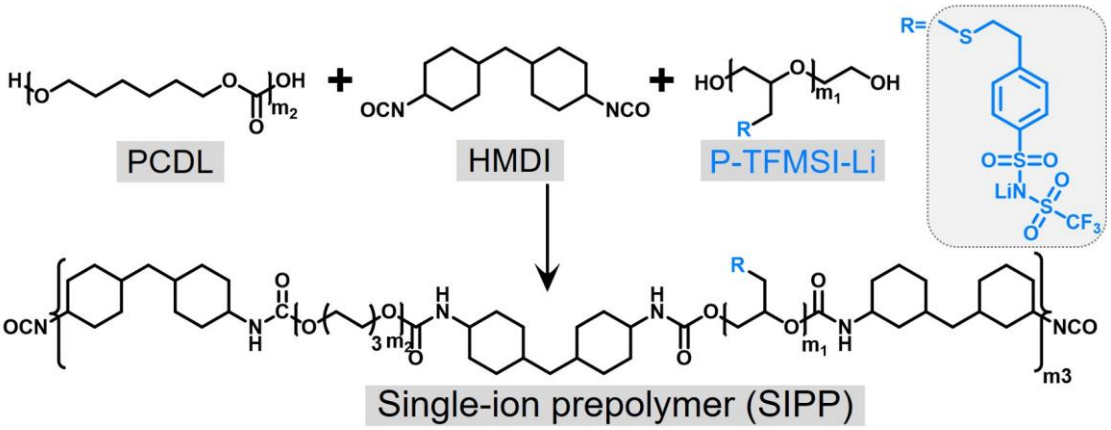

Figure S2. Synthetic route for single-ion prepolymer (SIPP).

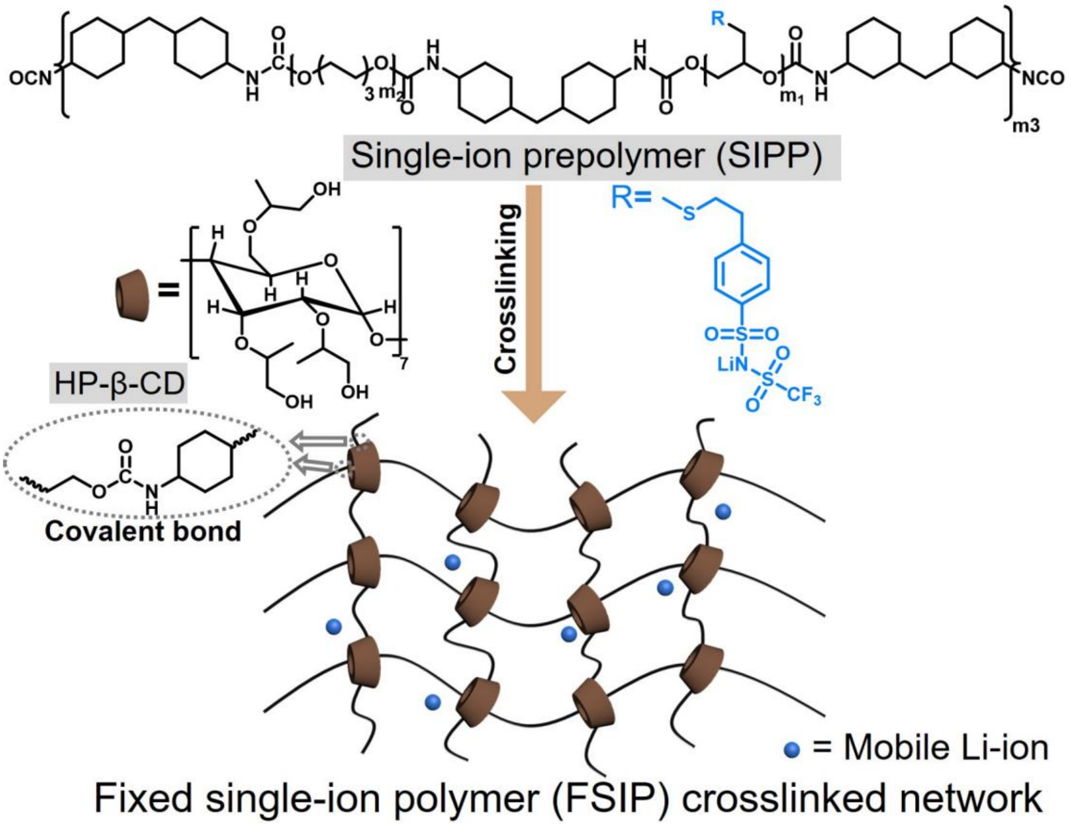

Figure S3. Synthesis schematic illustration and segment structure of fixed single-ion polymer (FSIP) crosslinked network.

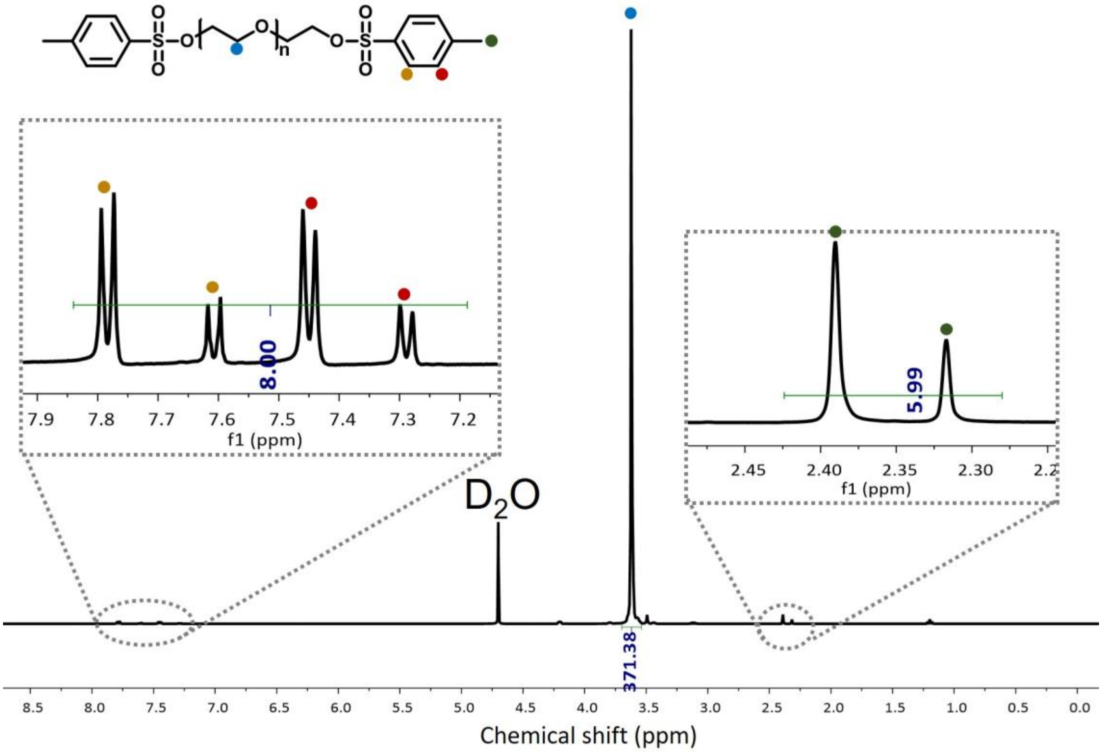

Figure S4. ^1H NMR spectrum of PEG-Ts (400 MHz, D_2O).

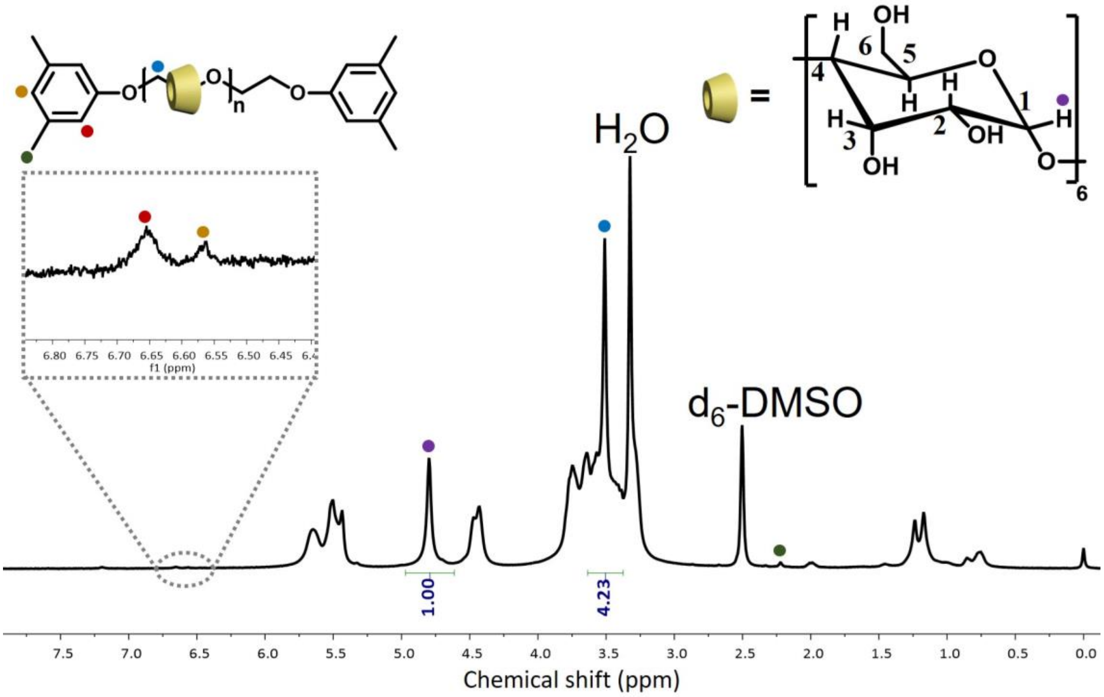

Figure S5. ^{1}H NMR spectrum of PR (400 MHz, d_6-DMSO).

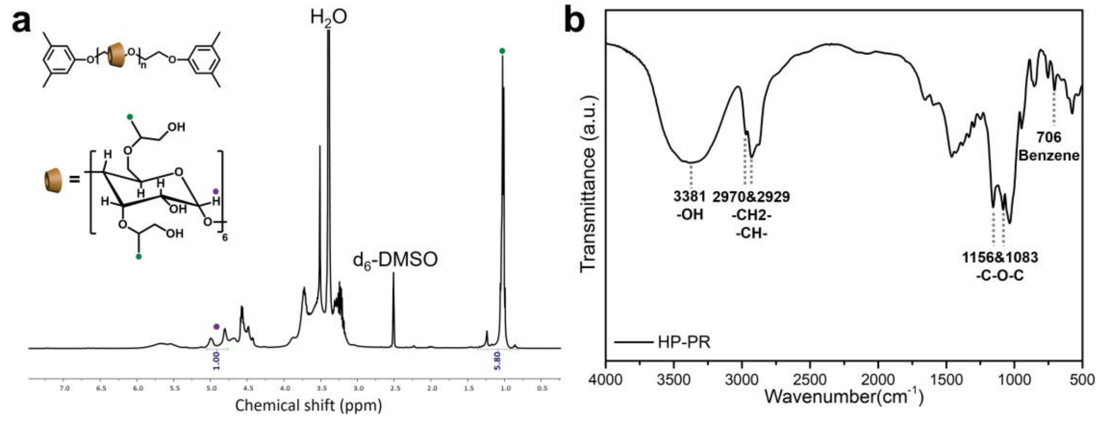

Figure S6. a) ${}^{1}H$ NMR spectrum of HP-PR (400 MHz, d_6-DMSO). b) FTIR spectrum of HP-PR.

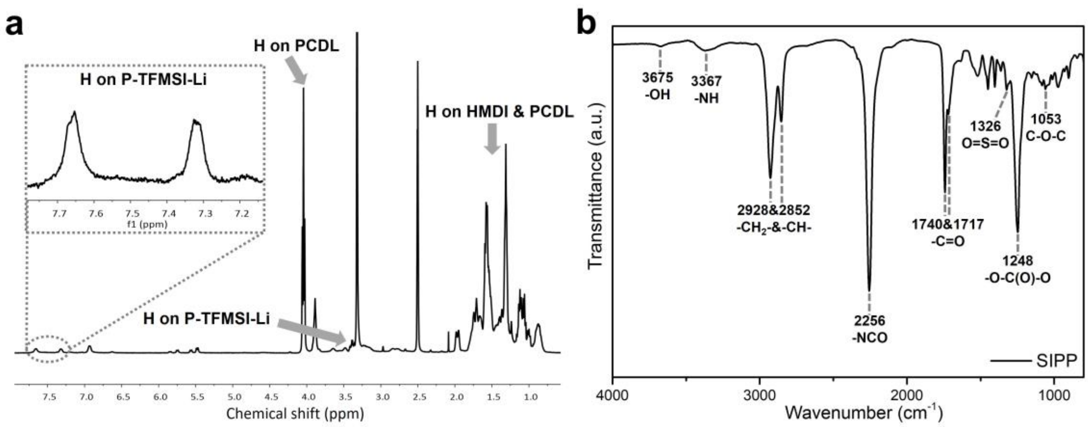

Figure S7. a) ^{1}H NMR spectrum of SIPP (400 MHz, d_6-DMSO). b) FTIR spectrum of SIPP. The strong absorption peak at 2256 cm^{-1} was assigned to a large quantity of unreacted NCO groups.

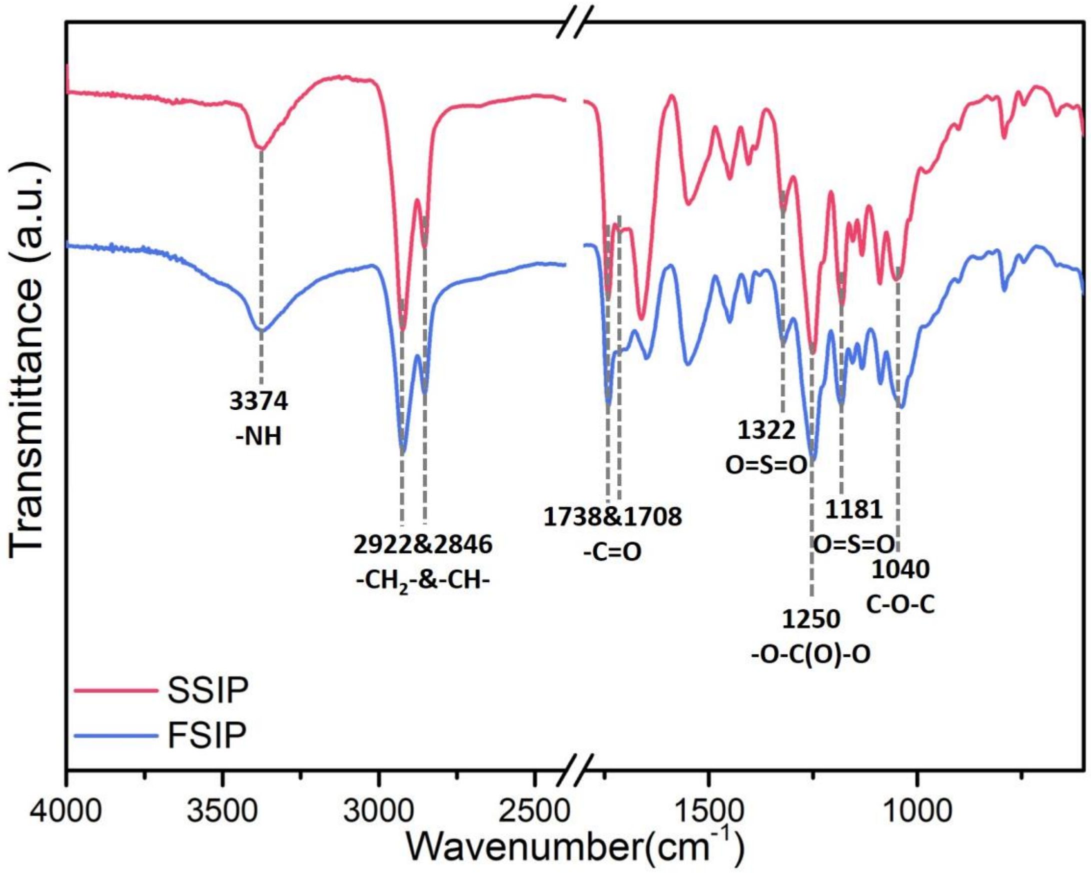

Figure S8. FTIR spectrum of SSIP and FSIP. The disappearence of the absorption peaks of -OH and -NCO proved that the reaction was completed.

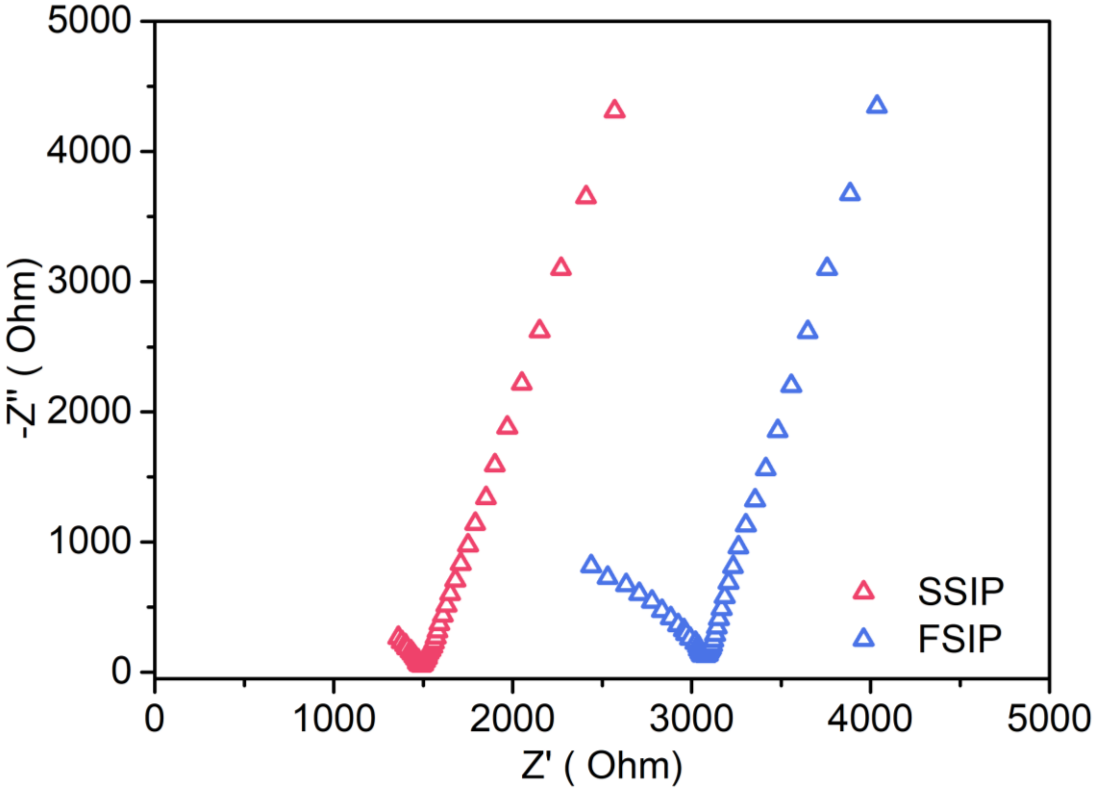

Figure S9. Electrochemical impedance spectra of SSIP and FSIP membranes under swollen state.

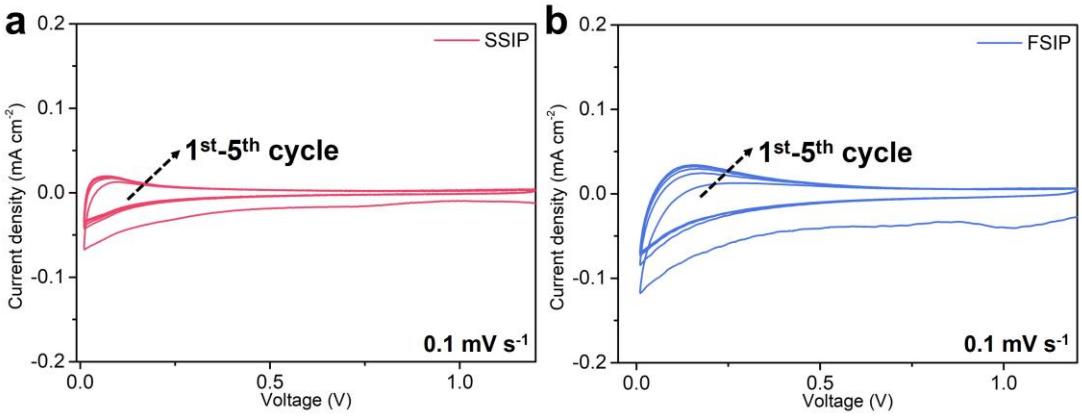

Figure S10. CV curves of electrodes composed by a) SSIP/CNT and b) FSIP/CNT. The scan rate was 0.1 mV s^{-1}. In the voltage range of 0.01 V - 1.2 V, few weak redox peaks appeared in the first scan, and the curves of the subsequent four circles were very stable and almost overlapped.

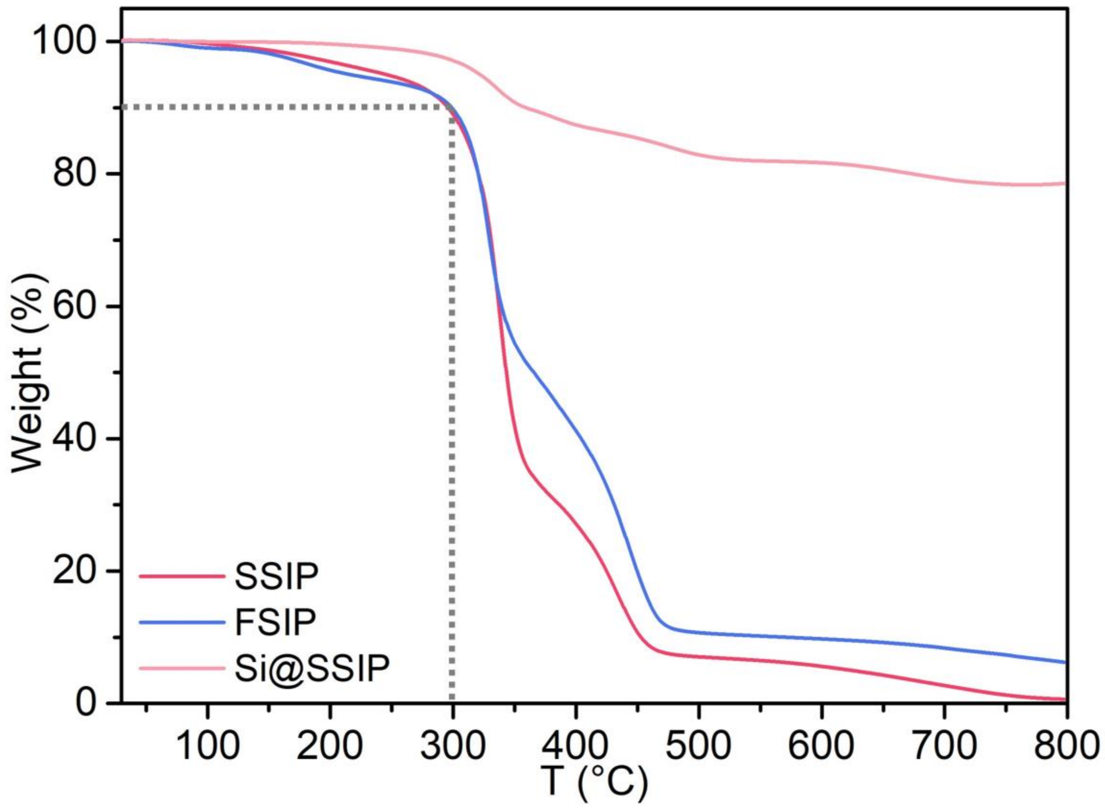

Figure S11. TGA curves of SSIP, FSIP and Si@SSIP (N_2 condition).

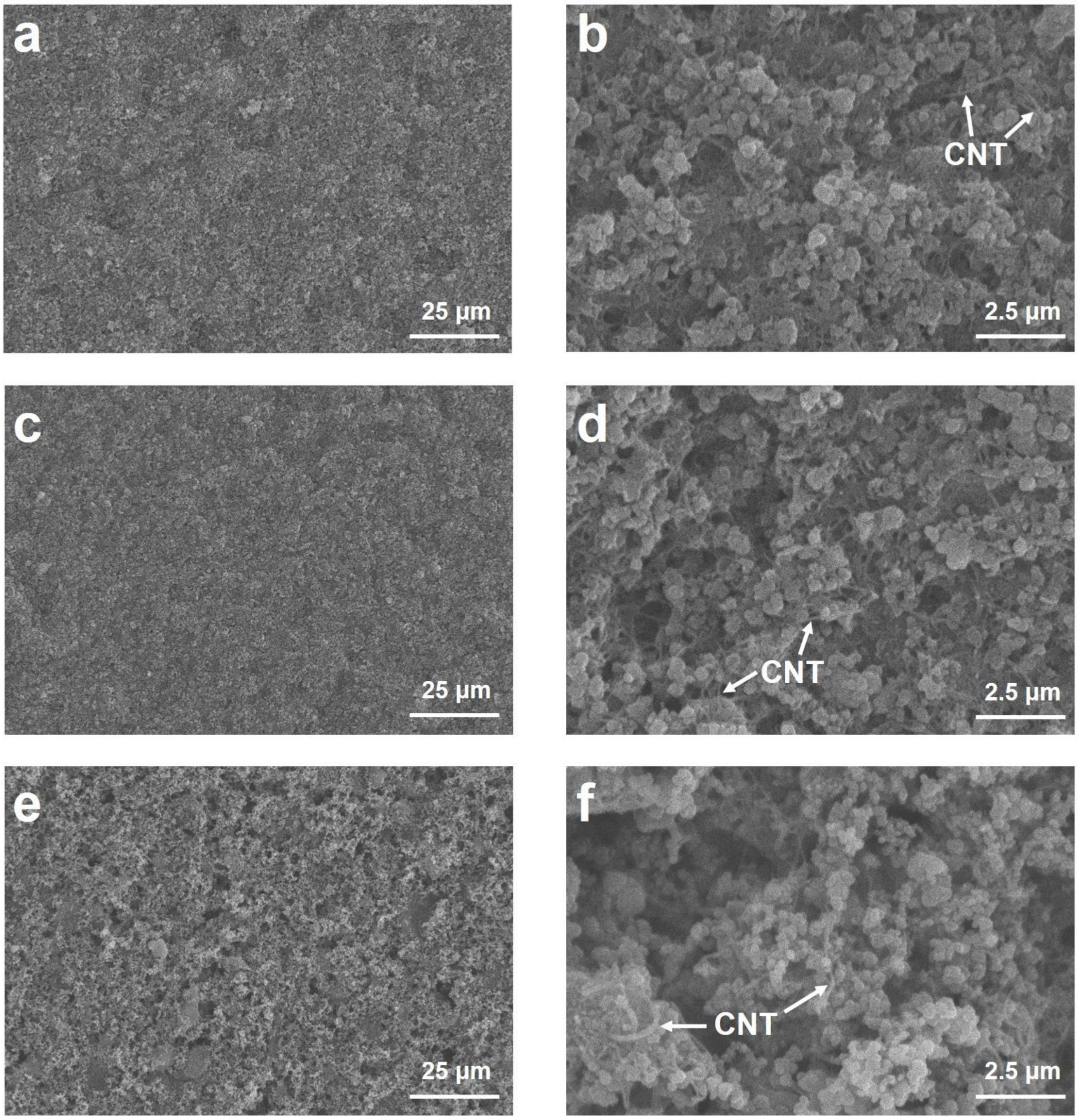

Figure S12. Top view SEM images of a), b) Si@SSIP c), d) Si@FSIP and e), f) Si@PVDF before cycling at different magnification.

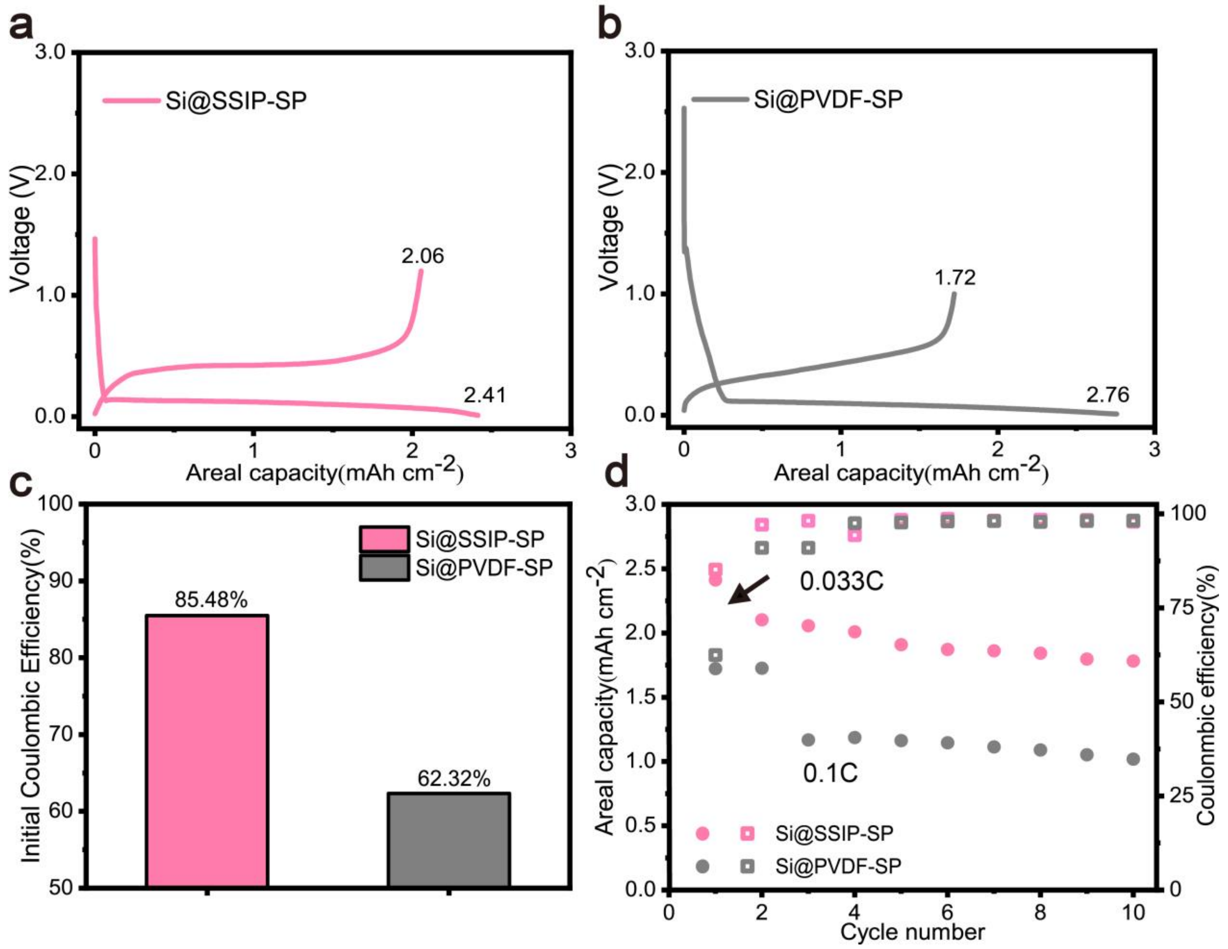

Figure S13. a) First charging and discharging curves of Si@SSIP-SP electrode at 0.033C. b) First charging and discharging curves of Si@PVDF-SP electrode at 0.033C. c) ICE of Si@SSIP-SP and Si@PVDF-SP electrodes. d) Cycling performance of Si@SSIP-SP and Si@PVDF-SP at 0.1C with first 2 activation cycles at 0.033C.

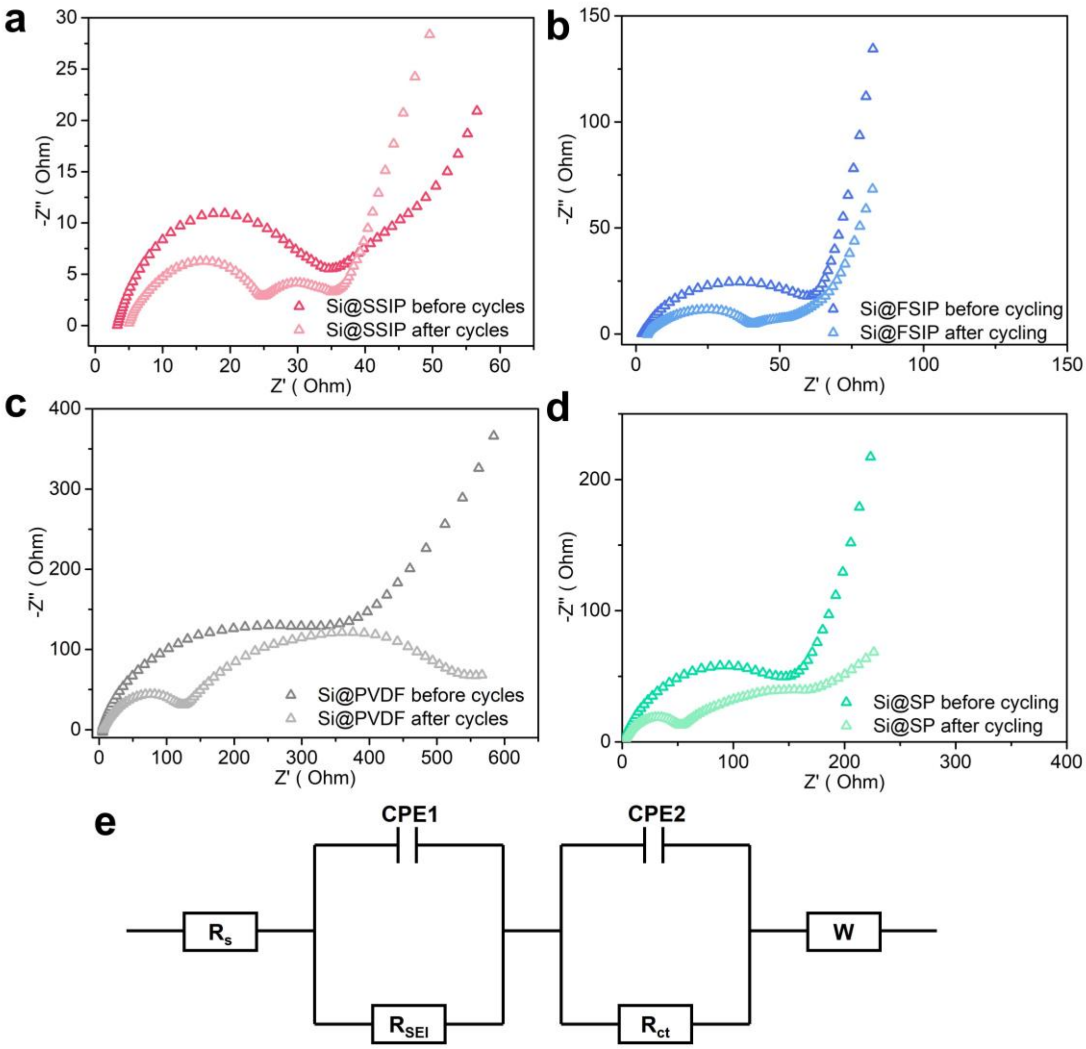

Figure S14. a-d) Electrochemical impedance spectra of Si electrodes with various binders before and after cycling. e) Relevant equivalent circuit diagram during EIS tests.

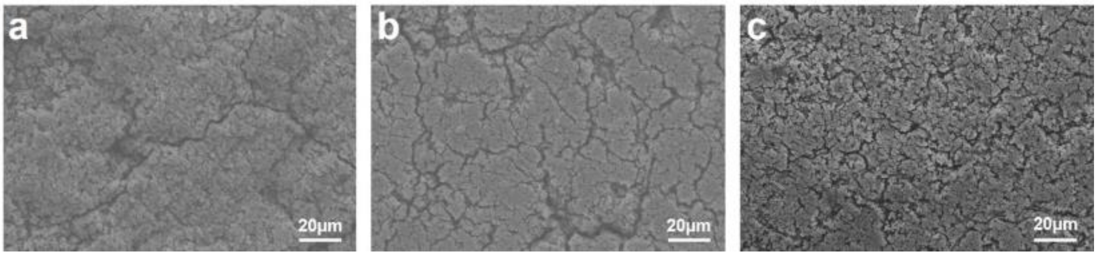

Figure S15. Top view SEM images of a) Si@SSIP, b) Si@FSIP and c) Si@PVDF after cycling. Lots of cracks appeared on the surface of the Si@FSIP and Si@PVDF electrodes and the electrodes had an obvious tendency of powdering. The morphology of the Si@SSIP electrode was relatively complete and only a few shallow cracks appeared on the surface.

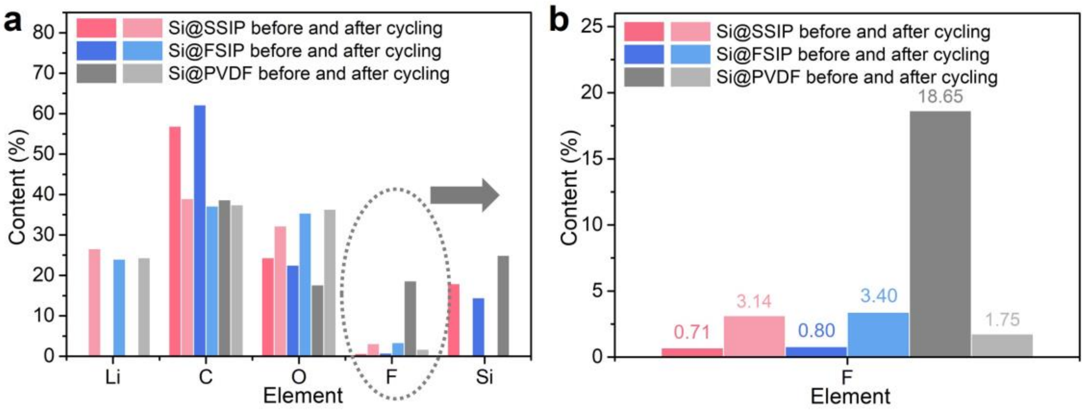

Figure S16. a) Various elements content and b) F element content on the surface of silicon electrodes with different binders before and after cycling. It was proved that SSIP and FSIP binders can effectively induce the formation of more LiF in SEI, and relatively fewer unstable components such as Li_2CO_3 and $LiOCO_2R$.

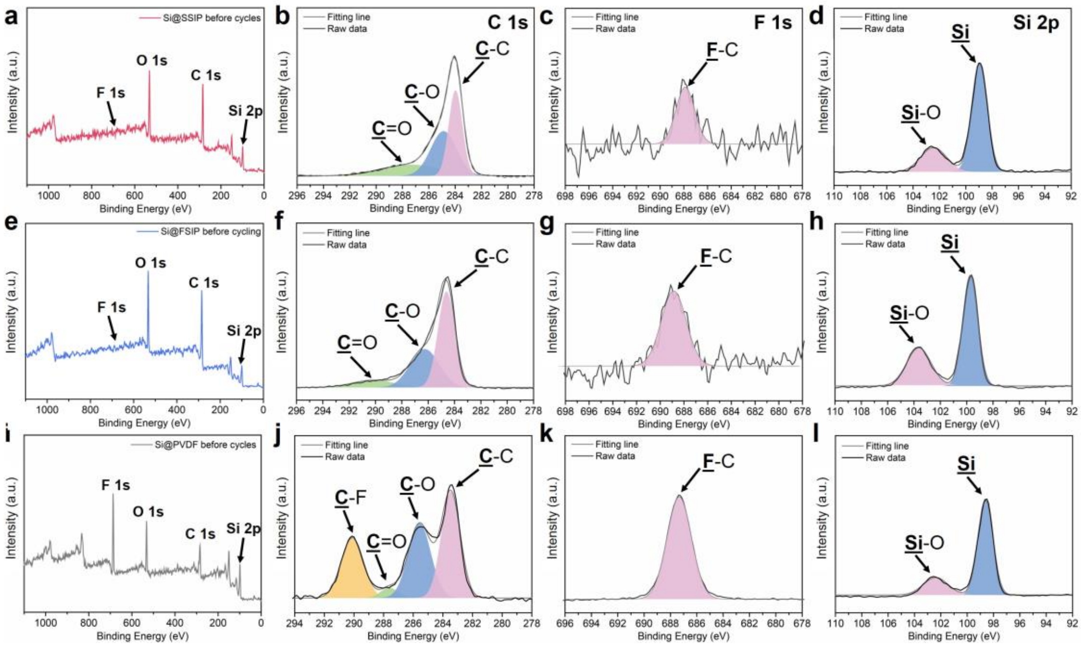

Figure S17. Full XPS spectra and high-resolution XPS spectra with related peaks fitting results of C 1s, F 1s and Si 2p of silicon electrodes with different binders before cycling: a-d) Si@SSIP, e-h) Si@FSIP, i-l) Si@PVDF.

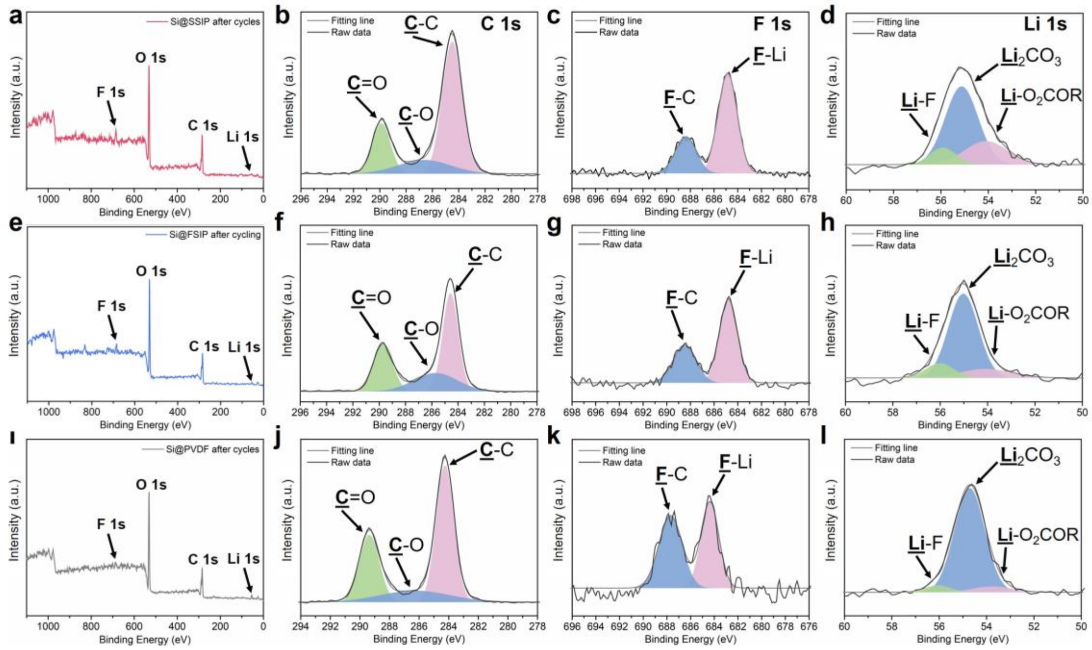

Figure S18. Full XPS spectra and high-resolution XPS spectra with related peaks fitting results of C 1s, F 1s and Si 2p of silicon electrodes with different binders after cycling: a-d) Si@SSIP, e-h) Si@FSIP, i-l) Si@PVDF.

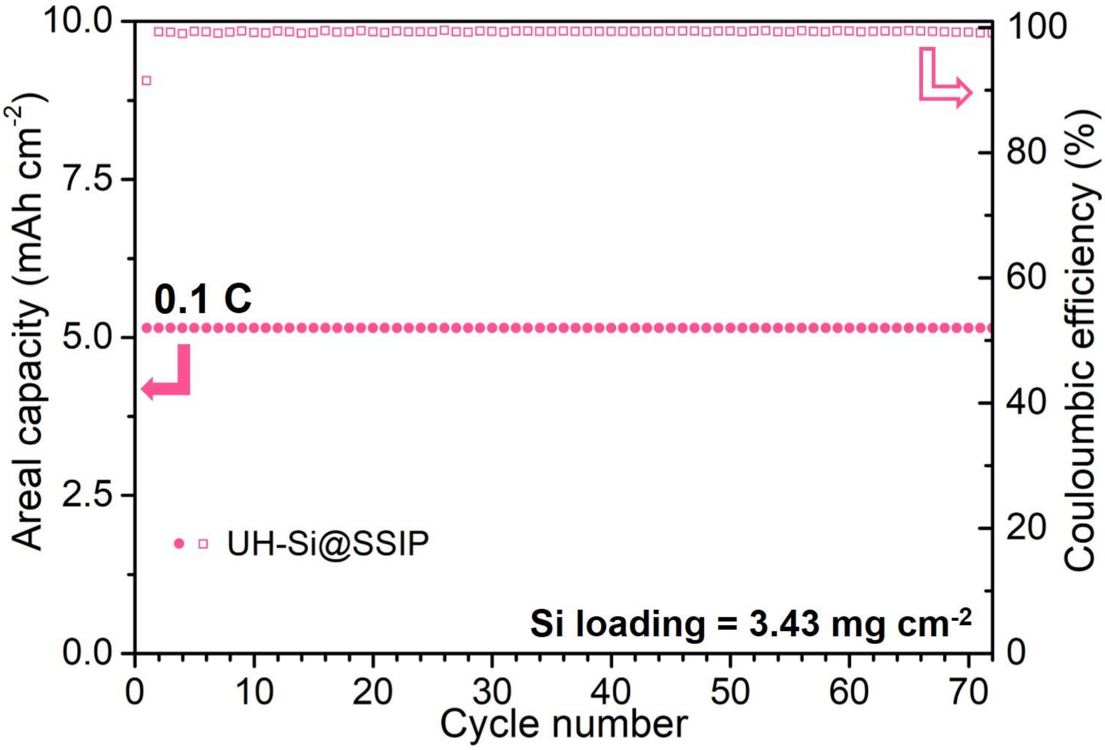

Figure S19. Areal discharge capacity and Coulombic efficiency of the UH-Si@SSIP electrodes with Si loading of 3.43 mg cm^{-2} at 0.1 C.

Table S1. Segment components of crosslinked polymer binders.

	HMDI	PCDL1k	P-TFSMI-Li	HP-PR[1]	HP-CD[1]
**SSIP**	0.3940 g<br>1.50 mmol	0.2500 g<br>0.25 mmol	0.1820 g<br>0.05 mml	0.1660 g<br>1.23 mmol	-
**FSIP**	0.3940 g<br>1.50 mmol	0.2500 g<br>0.25 mmol	0.1820 g<br>0.05 mml	-	0.1660 g<br>1.23 mmol
**SP**	0.3940 g<br>1.50 mmol	0.2500 g<br>0.25 mmol	-	0.1660 g<br>1.23 mmol	-

1. The number of moles calculation of multi-hydroxyl molecule was based on di-hydroxyl.

Table S2. R_{ct} and R_{SEI} of Si electrodes with various binders before and after cycling.

**Sample**	**Before cycling**		**After cycling**	
	**R_{ct}**	**R_{SEI}**	**R_{ct}**	**R_{SEI}**
**Si@SSIP**	33	-	18	23
**Si@FSIP**	65	-	24	38
**Si@PVDF**	545	-	468	130
**Si@SP**	188	-	182	56

Reference

[1] a) Y. Cai, C. Liu, Z. Yu, H. Wu, Y. Wang, W. Ma, Q. Zhang, X. Jia, *J. Power Sources* **2022,** *537*, 231478; b) Y. Cai, H. Wu, W. Yan, Z. Yu, W. Ma, C. Liu, Q. Zhang, X. Jia, *ACS Appl. Polym. Mater.* **2021,** *3*, 3254.

[2] a) R. Du, Z. Xu, C. Zhu, Y. Jiang, H. Yan, H. C. Wu, O. Vardoulis, Y. Cai, X. Zhu, Z. Bao, Q. Zhang, X. Jia, *Adv. Funct. Mater.* **2019,** *30*, 1907139; b) R. Du, Q. Jin, T. Zhu, C. Wang, S. Li, Y. Li, X. Huang, Y. Jiang, W. Li, T. Bao, P. Cao, L. Pan, X. Chen, Q. Zhang, X. Jia, *Small* **2022,** *18*, 2200533.
